# Prognostic awareness and its association with health outcomes in the last year of life

**DOI:** 10.1002/cam4.5286

**Published:** 2022-10-06

**Authors:** Semra Ozdemir, Isha Chaudhry, Sean Ng, Irene Teo, Chetna Malhotra, Eric Andrew Finkelstein, COMPASS Study Group

**Affiliations:** ^1^ Lien Centre for Palliative Care Duke‐NUS Medical School Singapore; ^2^ Signature Programme in Health Services and Systems Research Duke‐NUS Medical School Singapore; ^3^ Saw Swee Hock School of Public Health National University of Singapore Singapore; ^4^ National Cancer Centre Singapore Singapore; ^5^ Duke Global Health Institute Duke University Durham North Carolina USA

**Keywords:** anxiety, awareness, depression, policy, psychology, quality of life

## Abstract

**Background:**

Little research has examined changes in prognostic awareness (PA) in the last year of life and the extent PA change was associated with anxiety, depression, and spiritual well‐being among metastatic cancer patients.

**Methods:**

Two surveys were administered in the last year of life to 176 conveniently sampled Singaporean patients with stage 4 solid cancers. PA was assessed by asking patients whether they were aware that their treatments were unlikely to cure their cancer. Multivariable linear regression models were used to investigate the association of PA change with anxiety, depression, and spiritual well‐being.

**Results:**

The proportion of patients with accurate PA increased (39.2%–45.5%; *p* < 0.05) from the second‐last assessment to the last assessment before death. Those with inaccurate PA decreased (26.1%–20.4%; *p* < 0.05) while a third of patients remained uncertain at both assessments (34.7% and 34.1%). Compared to patients with inaccurate PA at both assessments, patients who reported accurate PA at both assessments reported worsened anxiety (*β* = 2.08), depression (*β* = 3.87), and spiritual well‐being (*β* = −4.45) while patients who reported being uncertain about their prognosis at both assessments reported worsened spiritual well‐being (*β* = − 6.30) at the last assessment before death (*p* < 0.05 for all).

**Conclusions:**

Interventions should dually focus on decreasing prognostic uncertainty at the end‐of‐life while minimising the psychological and spiritual sequelae associated with being prognostically aware. More research is needed to clarify the causes of prognostic uncertainty.

## Graphical abstract

Prognostic awareness and its association with helth outcomes in the last year of life.

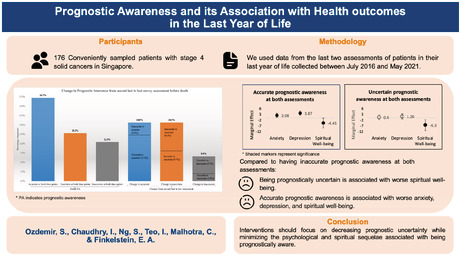

## 1 BACKGROUND

Prognostic awareness (PA) refers to the patient's understanding of their incurable disease and shortened life expectancy.^1^ The importance of PA has been highlighted in recent studies, with findings demonstrating associations between PA and informed decision‐making,^2^ reduction in futile treatments,^3^ and increased illness acceptance.^4^ Despite these benefits to the patient, concerns regarding the potential negative psychological consequences following prognostic disclosure have been raised,^5^ with research indicating that PA can be associated with worse patient outcomes, including anxiety,^6^ depression,^7^ and spiritual^8^ well‐being. These results can be generally understood to be in line with the conceptual framework provided by the Prognostic Awareness Impact Scale (PAIS), which posits that prognostically aware patients, if they do not have appropriate adaptive mechanisms, can suffer negative psychological consequences.^9^ However, the majority of these studies have been cross‐sectional—little literature has examined how PA can change over time and how this evolution is associated with patient psychological and spiritual well‐being.

While a literature search found six longitudinal studies^7^, ^10^, ^11^, ^12^, ^13^, ^14^ that focused on the associations of PA with our outcomes of interest (psychological and spiritual well‐being), consensus remain lacking. Though PA was correlated with improved depression in one study,^14^ others indicated that being prognostically aware was associated with worse depression^7^, ^10^, ^12^ and spiritual well‐being.^13^ Additionally, despite prior cross‐sectional evidence,^6^, ^15^, ^16^, ^17^, ^18^ none of the longitudinal studies examined reported significant associations between PA and anxiety.^7^, ^10^, ^11^, ^12^ Several methodological limitations with extant longitudinal research also remain. First, most studies dichotomised PA into binary aware/unaware categories,^7^, ^10^, ^11^, ^13^, ^14^ an approach that may have excluded individuals who were uncertain or unsure about their prognoses. Only one longitudinal study specifically focused on how PA changed over time.^13^

The aim of this study was therefore to examine: (1) how PA (defined in this study as awareness of treatment intent) changed between two assessments in the last year of life among patients with metastatic cancer and (2) the associations between PA change and anxiety, depression, and spiritual well‐being at the last assessment before death. We hypothesised that compared to maintaining inaccurate PA (at both assessments), maintaining accurate/uncertain PA (at both assessments) or switching between PA states (accurate, inaccurate, or uncertain) would be associated with worse anxiety, depression, and spiritual well‐being before death.

## 2 METHODS

### 2.1 Participants and study settings

Data for this study were obtained from the Cost of Medical Care of Patients with Advanced Serious Illness in Singapore (COMPASS) project, a prospective cohort study of patients with stage IV solid cancer.^19^ Eligible participants were recruited from outpatient medical oncology clinics in two major public Singaporean hospitals. The eligibility criteria included being: (1) ≥21 years old; (2) a Singaporean citizen/permanent resident; (3) cognitively able to provide informed consent (i.e., determined using medical records or an Abbreviated Mental Test^20^ for participants ≥60 years old); (4) diagnosed with stage IV solid malignancy; (5) having an Eastern Cooperative Oncology Group performance score of ≥2.^21^ The questionnaires were administered by trained interviewers in the participant's language of choice (English, Mandarin, and Malay). Eligible patients were administered the baseline questionnaire between July 2016 and March 2018. They were then surveyed every 3 months until death. The PA question was asked every 9 months to reduce cognitive burden on the respondents. More information on the study protocol can be found elsewhere.^19^ Ethical IRB approval was given by the SingHealth Centralised Institutional Review Board for the main COMPASS study (2015/2781). Informed consent was provided by all participants.

This study utilised data from the last two assessments (that the PA question was asked) of patients in their last year of life (± a few months) and who died between July 2016 and May 2021. We only included those who answered at least two surveys within the last year of their life to investigate the evolution of PA between two assessments and how it was associated with patient outcomes at the last assessment before death.

### 2.2 Measures

#### 2.2.1 Prognostic awareness

PA was examined using the statement “The current treatments you are taking for your illness will cure you” Responses included “yes”, “no” or “not sure”. “No” responses were considered as having *accurate* PA, “Yes” responses were considered as having *inaccurate* PA, and “Not Sure” responses were considered as having *uncertain* prognostic awareness. This type of question has been very commonly used to assess PA among cancer patients.^1^, ^22^, ^23^, ^24^

#### 2.2.2 Anxiety and Depression

Anxiety and depression were assessed with the Hospital Anxiety and Depression Scale (HADS). Each subscale (anxiety and depression) consisted of 7‐items scored on a Likert scale (0 to 3), where higher scores on each subscale reflected worse anxiety or depression.^25^

#### 2.2.3 Spiritual well‐being

Spiritual well‐being was assessed using the Functional Assessment of Chronic Illness Therapy – Spiritual Well‐Being (FACIT‐SP) scale, a 12‐item questionnaire examining two components of spiritual well‐being (meaning, peace and faith) among cancer patients. Items were scored on a Likert scale (0–4). Total score ranged between 0 and 48 with a higher score indicating better spiritual well‐being.^26^

All scales had high internal consistency reliability (Cronbach alpha of at least 0.78; anxiety = 0.78, depression = 0.82, spiritual well‐being = 0.85).

#### 2.2.4 Patient characteristics

Patients reported their age, gender, and level of education.

### 2.3 Statistical Analyses

We investigated PA at each assessment and change in PA between the last two assessments before patients' death. Change in PA was categorised into the following categories: (i) maintaining accurate PA (at both assessments), (ii) maintain inaccurate PA (at both assessments), (iii) maintaining uncertain PA (at both assessments), (iv) changed to accurate PA, that is, changed from inaccurate or uncertain PA to accurate PA at the last survey assessment, and (v) changed from accurate PA to inaccurate or uncertain PA at the last survey assessment. *p*‐values ≤0.05% were considered significant.

We performed analysis of variance test to investigate whether patient outcomes (anxiety, depression, and spiritual well‐being) were different based on their PA state at each assessment. Finally, we conducted multivariable linear regression models to investigate the association of change in PA (independent variables) with each patient outcome: anxiety, depression, and spiritual well‐being at the last assessment before death. Further, as a sensitivity analysis we examined these associations when depression and anxiety outcomes were specified as categorical variables using logistic regression. We categorised patients as borderline abnormal or abnormal if their individual HADS depression or anxiety score was ≥8.

The models controlled for patient age, gender, and level of education (high school or below vs above high school). We used Stata 16.1 for all analyses.

## 3 RESULTS

### 3.1 Sample characteristics

We recruited 600 eligible patients in the COMPASS study. Of these, 55 patients (9.1%) had dropped out of the study during the study period while 412 (68.7%) patients had died by 31 May 2021. Out of these, 22 patients were excluded as they had not responded to the question on prognostic awareness. Out of 390 patients, 176 patients answered the question on PA at least two times during their last year of life and consisted of our final analytical sample. Average (standard deviation [SD]) time between the last survey assessment and patient death was 6.8 (3.7) months, and time between the second last and last survey assessment was 8.6 (1.0) months.

Sample characteristics are described in Table 1. At the end‐of‐life, mean (SD) age was 61.8 (10.0) years. Nearly half our participants were female (48.3%) and nearly one‐third (32.4%) had above high school education. The majority of the participants were Chinese (79.5%). There was a significant increase in mean anxiety (2.6 ± 2.8 versus 4.0 ± 3.8) and depression (3.2 ± 3.2 versus 4.7 ± 4.2) scores and a significant decline in spiritual well‐being (38.5 ± 7.3 versus 35.5 ± 8.5) scores between the last two assessments (*p* < 0.01 for all).

**TABLE 1:** Descriptive characteristics (*n* = 176)

Age at last survey assessment, mean (SD)	61.8 (10.0)
Female, *n* (%)	85 (48.3)
Education, *n* (%)	
High school or below	119 (67.6)
Above high school	57 (32.4)
Chinese, *n* (%)	140 (79.5)
Prognostic awareness, *n* (%)	
At the second last assessment	
Accurate	69 (39.2)
Inaccurate	46 (26.1)
Uncertain	61 (34.7)
At the last assessment	
Accurate	80 (45.5)
Inaccurate	36 (20.4)
Uncertain	60 (34.1)
Anxiety, mean (SD)^a^	
At the second last assessment	2.6 (2.8)
At the last assessment	4.0 (3.8)
Depressive symptoms, mean (SD)^a^	
At the second last assessment	3.2 (3.2)
At the last assessment	4.7 (4.2)
Spiritual well‐being, mean (SD)^a^	
At the second last assessment	38.5 (7.3)
At the last assessment	35.5 (8.5)

^a^ *t*‐test indicating *p* < 0.01 for difference between the last two assessments before death.

### 3.2 Prognostic awareness

Overall, the proportion of patients who reported accurate PA increased from second‐last (39.2%) to the last survey assessment before death (45.5%). The proportion of patients who reported inaccurate PA decreased (26.1% and 20.4%) while about one‐third of patients reported being uncertain about their prognosis at both assessments (34.7% and 34.1%) (Table 1). A chi‐squared test showed that the differences in PA levels between the last two assessments were significant (*p* < 0.05).

**FIGURE 1:**
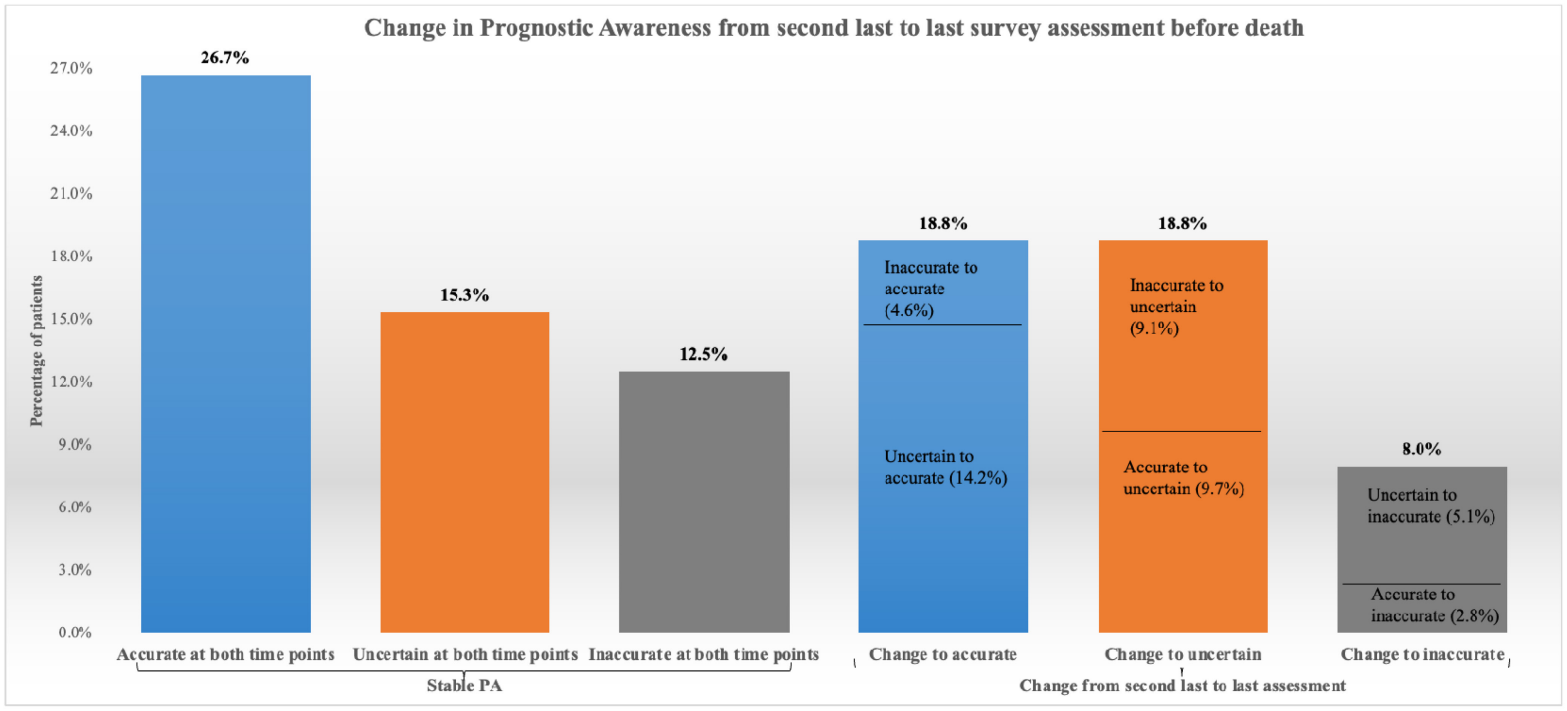
Prognostic awareness in the last two assessments before death (*n* = 176)

Over one‐fourth of the patients (26.7%) reported maintaining accurate PA while 12.5% reported maintaining inaccurate PA and 15.3% reported maintaining uncertain PA at both assessments (Figure 1). About a fifth (18.8%) of the patients changed (from uncertain or inaccurate PA) to having accurate PA and another 18.8% changed (from accurate or inaccurate PA) to being uncertain about their prognosis at the last assessment while 8% of the patients changed (from uncertain or accurate PA) to having inaccurate PA.

### 3.3 Prognostic awareness and patient outcomes at each assessment

**FIGURE 2:**
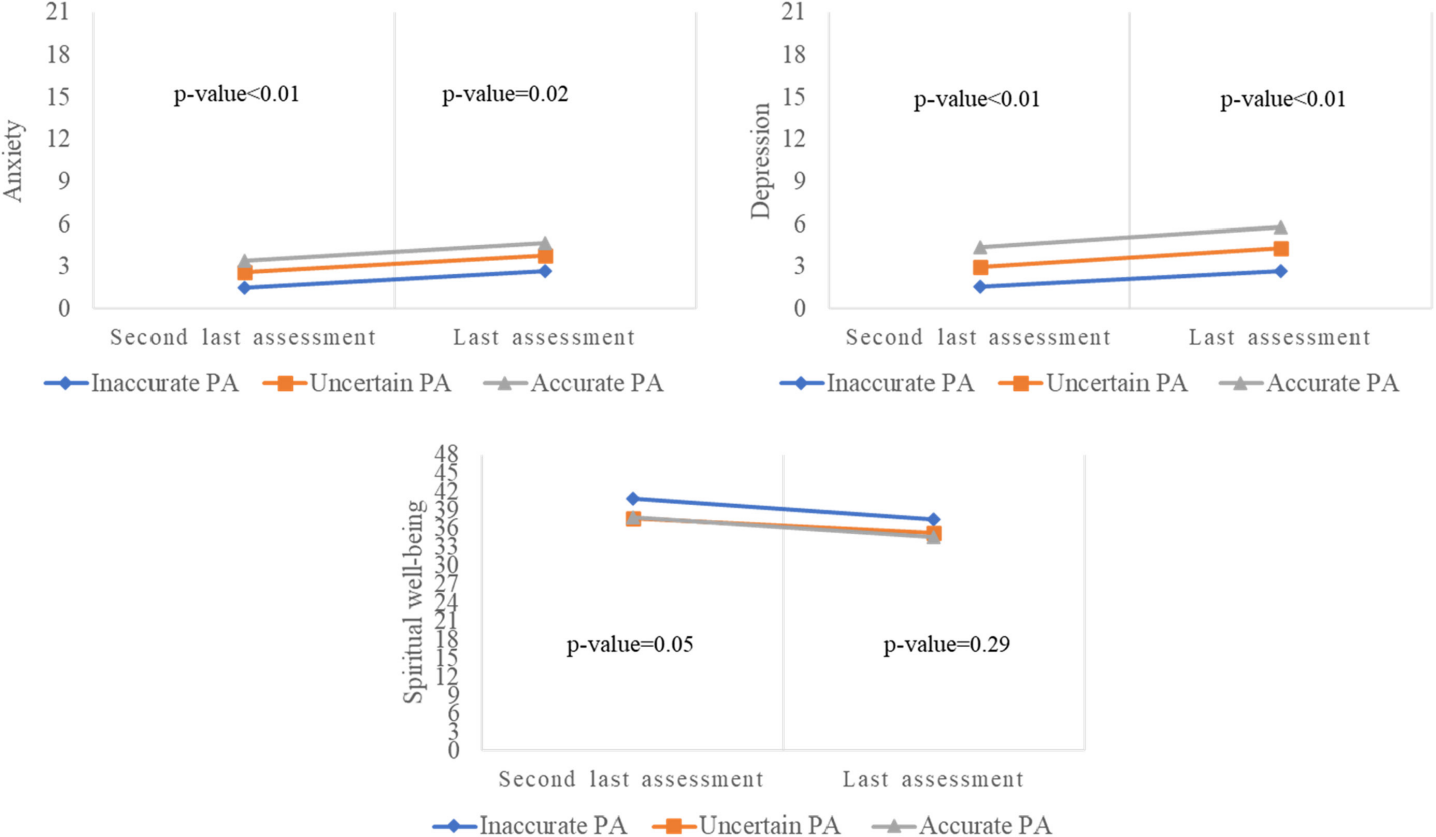
Patient outcomes for patients with accurate PA, uncertain PA or inaccurate PA in the last two assessments before death (*n* = 176)

Patients with accurate PA reported the highest anxiety and depression scores at each assessment while those with inaccurate PA reported the lowest anxiety and depression scores at each assessment (Figure 2). The differences in mean anxiety and depression scores between the patient groups (with different PA levels) in each assessment were significant (*p* < 0.05). No significant difference was observed for the mean spiritual well‐being score among patients with different levels of PA at each assessment (*p* ≥ 0.05).

### 3.4 Associations of change on prognostic awareness with patient outcomes at the end‐of‐life

In the multivariable analyses, patients who maintained inaccurate PA at both assessments were used as the reference group. Patients who maintained accurate PA at both assessments reported higher anxiety (*β* [95% confidence interval]: 2.08 [0.15, 4.00], *p*‐value: 0.03) and higher depression (3.87 [1.79, 5.96], *p* < 0.01) scores, and worse spiritual well‐being (−4.45 [−8.82, −0.08], *p*‐value: 0.05) scores (Table 2). Patients who maintained uncertain PA at both assessments reported worse spiritual well‐being (−6.30 [−11.10, −1.51], *p*‐value: 0.01) score at the last assessment.

**TABLE 2:** Associations of change in level of prognostic awareness with patient end‐of‐life outcomes (*n* = 176)

	Anxiety	Depression	Spiritual well‐being
Prognostic awareness (ref. inaccurate at both assessments)			
Accurate at both assessments			
Coefficient	2.08**	3.87***	−4.45**
95% CI	0.15, 4.00	1.79, 5.96	−8.82, −0.08
*p*‐value	0.03	<0.01	0.05
Uncertain at both assessments			
Coefficient	0.40	1.26	−6.30**
95% CI	−1.71, 2.51	−1.03, 3.54	−11.10, −1.51
*p*‐value	0.71	0.28	0.01
Changed to accurate			
Coefficient	1.25	1.99*	−4.13*
95% CI	−0.80, 3.29	−0.22, 4.20	−8.78, 0.51
*p*‐value	0.23	0.08	0.08
Changed to inaccurate/uncertain			
Coefficient	0.68	0.83	−3.23
95% CI	−1.25, 2.60	−1.25, 2.92	−7.61, 1.14
*p*‐value	0.49	0.43	0.15
Age			
Coefficient	−0.06**	−0.07**	0.03
95% CI	−0.12, −0.04	−0.13, −0.005	−0.10, 0.16
*p*‐value	0.04	0.04	0.66
Gender (ref: Male)			
Female			
Coefficient	0.57	−0.74	2.56*
95% CI	−0.59, 1.73	−2.00, 0.52	−0.09, 5.21
*p*‐value	0.33	0.25	0.06
Education (ref: High school or below)			
Above high school			
Coefficient	−0.33	−1.12*	2.12
95% CI	−1.55, 0.90	−2.44, 0.20	−0.66, 4.90
*p*‐value	0.60	0.10	0.13

*Note*: ***, ** and * indicates significance at the 1%, 5% and 10% level, respectively.

The sensitivity analysis showed similar findings. Compared to those who had inaccurate PA at both assessments, patients with accurate PA at both assessments were more likely to report borderline abnormal or abnormal depression (Odds ratio [95% CI]: 4.82 [1.19, 19.49], *p*‐value = 0.03). We did not find any significant associations with anxiety (Table S1).

## 4 DISCUSSION

This longitudinal study aimed: (1) to examine how PA changed between two assessments in the last year of life among patients with metastatic cancer and; (2) to investigate the associations between PA change and anxiety, depression, and spiritual well‐being at the last assessment before death.

Lending support to prior literature,^11^ results indicated that the overall proportion of participants who reported accurate PA increased from 39% to 46% during their last year of life. A possible reason for these results is that patients were able to observe their increasing symptoms and declining health and therefore had more insight into their prognoses. These results are encouraging as they suggest that patients become increasingly aware of their prognosis near death. However, we also found that over 20% and 34% of patients respectively reported inaccurate and uncertain PA in their last few months of life. Worryingly, 2.8% of patients changed from reporting accurate to inaccurate PA while 9.7% changed from reporting accurate to uncertain PA. While it is possible that these patients may have been in denial,^27^ were avoiding discussing (or thinking) about the eventuality for fear of hastening it^28^ or were trying to maintain hope,^29^ these are concerning findings as they indicate that a number of patients may be holding on to unrealistic beliefs of curability even when close to death.

Our findings that patients who maintained accurate PA at both assessments (compared to those who maintained consistently inaccurate PA) reported higher depression and lower spiritual well‐being scores are consistent with prior longitudinal studies^7^, ^10^, ^12^, ^13^ and similar to previously discussed thresholds for minimally clinically important differences for the HADS and FACT‐G subscales.^30^, ^31^ However, contrary to the non‐significant findings of past literature, we also found that patients maintaining consistently accurate PA reported higher anxiety similar to the clinically important differences found in the HADS.^30^ The reason for this discrepancy is unclear but may be due to methodological differences— we examined the evolution of PA through two different time‐points, an approach which allowed for a better longitudinal understanding of PA evolution. We also found that patients with uncertain PA at both assessments reported lower spiritual well‐being compared to those who had inaccurate PA at both assessments. This may be because patients who are uncertain/unsure of their prognosis may not be able to adequately prepare themselves for the inevitable, leading to existential (and/or spiritual) distress. These results are important given that over one‐third of our sample reported having uncertain PA before they died. Clinically, they suggest that a significant portion of patients who were ‘uncertain’ may not be garnering the benefits of being prognostically aware yet may be excluded from interventions targeting patients who reported inaccurate PA. In all, while this study shows that there are psychological risks to being prognostically aware, it is important that our results are not taken as support to withhold diagnoses. Instead, they should be taken as evidence that patients (especially those at the end‐of‐life) require further psychological assistance. To facilitate this, clinicians can be provided additional training so that they will be better able to deliver complex and sensitive clinical information to patients in an understandable manner.

The main strengths of this study included our examination of PA evolution in the last year of life among metastatic cancer patients and our examination of patients who were uncertain of their PA in their last year of life. Our findings serve to improve our theoretical understanding regarding evolution of PA and contribute to sparse (but growing) longitudinal research examining the associations between PA and important quality of life dimensions. Clinically, our results lend support to research findings promoting the fostering of PA. Specifically, they suggest that while initial fears regarding the psychological sequelae associated with accurate PA may be justified, it may also be worse for the patient psyche if their disease status was left uncertain or unclear. These findings can have important implications given that a substantial proportion of patients (over a fifth in our sample) can became increasingly uncertain nearer to death. This means that current interventions promoting PA should not only target those with inaccurate PA, but also be expanded to capture patients who are unsure about their prognosis.

Our results should be taken in the context of several limitations. First, as this study was correlational, no conclusions regarding directionality can be drawn. Given that our participants consisted specifically of Singaporean metastatic cancer patients, results may also not be representative of other countries or terminal illnesses. Secondly, future research should also consider the inclusion of measures that specifically examine death anxiety. This would allow for a better understanding of how patients perceive their impending end. The focus of this study was also solely on a specific dimension of PA (i.e., curability) and its longitudinal associations with anxiety, depression, and spiritual well‐being. While our study contributes to the sparse research on the evolution of PA and psychological outcomes over time, future research should expand on our findings with the inclusion of multiple PA dimensions (i.e., awareness of life expectancy or advanced/terminal cancer). Research can also investigate the confounding variables such as pre‐existing mental illness or acceptance of prognosis on the associations between PA and the noted outcomes. Lastly, while practicality limited to our analyses to specific psychological dimensions, future studies should consider longitudinally examining the associations between PA and other variables important to the end‐of‐life experience including acceptance^16^ and peace.^32^ They can also use qualitative interviews to understand why patients with accurate PA would become confused of their prognosis.

In conclusion, through our longitudinal examination of PA patterns among metastatic cancer patients in their last year of life, we found that a significant number of patients reported inaccurate or uncertain PA even when close to death. Our findings suggest that interventions should target to improve uncertainty and confusion regarding prognosis, and must be accompanied by efforts to improve the psychological, and spiritual well‐being of patients. This would enable patients to obtain the benefits of PA while diminishing the accompanying psychological sequelae.

## AUTHOR CONTRIBUTIONS

**Semra Ozdemir:** Conceptualization (equal); formal analysis (equal); investigation (equal); methodology (equal); project administration (equal); supervision (equal); writing – original draft (equal); writing – review and editing (equal). **Isha Chaudhry:** Formal analysis (equal); writing – original draft (equal); writing – review and editing (equal). **Sean Ng:** Writing – original draft (equal); writing – review and editing (equal). **Irene Teo:** Methodology (equal); project administration (equal); writing – review and editing (equal). **Chetna Malhotra:** Methodology (equal); project administration (equal); writing – review and editing (equal). **Eric Finkelstein:** Supervision (equal); writing – review and editing (equal).

## FUNDING INFORMATION

Financial support for this study was provided by Singapore Millennium Foundation (2015‐SMF‐0003) and Lien Centre for Palliative Care (LCPC‐IN14‐0003). The funding agreement ensured the authors' independence in designing the study, interpreting the data, writing, and publishing the report.

## CONFLICT OF INTEREST

The authors report no conflicts of interest.

## Supporting information


Table S1.

## Data Availability

The data that support the findings of this study are available from the corresponding author upon reasonable request.
