# Supplementary material for: Prognostic awareness and its association with health outcomes in the last year of life
Source: Cancer Med. 2022 Oct 6;12(4):4801–8. doi: 10.1002/cam4.5286 (PMC9972138; doi:10.1002/cam4.5286)
Supplement: Supplementary file 1 — Table S1. [file CAM4-12-4801-s001.docx]

**Supplementary Material, Table S1. Sensitivity analysis: Associations of change in level of prognostic awareness with patient end-of-life psychological outcomes, n=176**

|  | | **Borderline abnormal or abnormal** | |
| --- | --- | --- | --- |
|  | | **Anxiety** | **Depression** |
| Prognostic awareness (ref. inaccurate at both assessments) | |  |  |
| Accurate at both assessments | Odds ratio | 2.05 | 4.82*** |
|  | 95% CI | 0.50, 8.43 | 1.19, 19.49 |
|  | p-value | 0.32 | 0.03 |
| Uncertain at both assessments | Odds ratio | 1.06 | 1.42 |
|  | 95% CI | 0.20, 5.49 | 0.29, 6.88 |
|  | p-value | 0.95 | 0.66 |
| Changed to accurate | Odds ratio | 1.81 | 2.47 |
|  | 95% CI | 0.41, 7.99 | 0.56, 10.81 |
|  | p-value | 0.43 | 0.23 |
| Changed to inaccurate/uncertain | Odds ratio | 0.92 | 1.37 |
|  | 95% CI | 0.20, 5.49 | 0.31, 6.88 |
|  | p-value | 0.91 | 0.67 |

Models were controlled for age, gender and education.
